# Supplementary material for: A Core Transcription Regulatory Circuitry Defining Microglia Cell Identity Inferred from the Reanalysis of Multiple Human Microglia Differentiation Protocols
Source: Brain Sci. 2021 Oct 11;11(10):1338. doi: 10.3390/brainsci11101338 (PMC8533937; doi:10.3390/brainsci11101338)
Supplement: Supplementary file 1 [file brainsci-11-01338-s001.zip › Supplementary_figures_Mendoza.pdf]

A core transcription regulatory circuitry defining microglia cell identity inferred from the reanalysis of multiple microglia differentiation protocols

Antoine Aubert[1], François Stüder[1], Bruno Maria Colombo[1], and Marco Antonio Mendoza-Parra[1,*]

[1] UMR 8030 Génomique Métabolique, Genoscope, Institut François Jacob, CEA, CNRS, University of Evry-val-d'Essonne, University Paris-Saclay, 91057 Évry, France.

*Corresponding author: Marco Antonio Mendoza-Parra

E-mail: mmendoza@genoscope.cns.fr / Phone: +33 (0) 1 60 76 42 92

Table of contents

Supplementary Figure S1 : Step by Step visualization of RNA-seq readouts on QC Genomics platform.

Supplementary Figure S2: Volcano plots revealing the differentially expressed genes (DEGs) in iMGL datasets generated by the various differentiation protocols.

Supplementary Figure S3. Master regulatory Transcription factors predicted by TETRAMER for each of the differential expression data associated to each of the iMGL differentiation protocols.

Supplementary Table S1 : Public Datasets and their associated identifiers (GSM) used in this comparative study (Excel file).

Supplementary Table S2: Differential gene expression readouts (DeSeq2) (Excel file).

Supplementary Table S3: Microglia-specific up-regulated genes identified in this study (Excel file).

Supplementary Table S4: Master regulatory indexes per TF assessed by TETRAMER (Excel file).

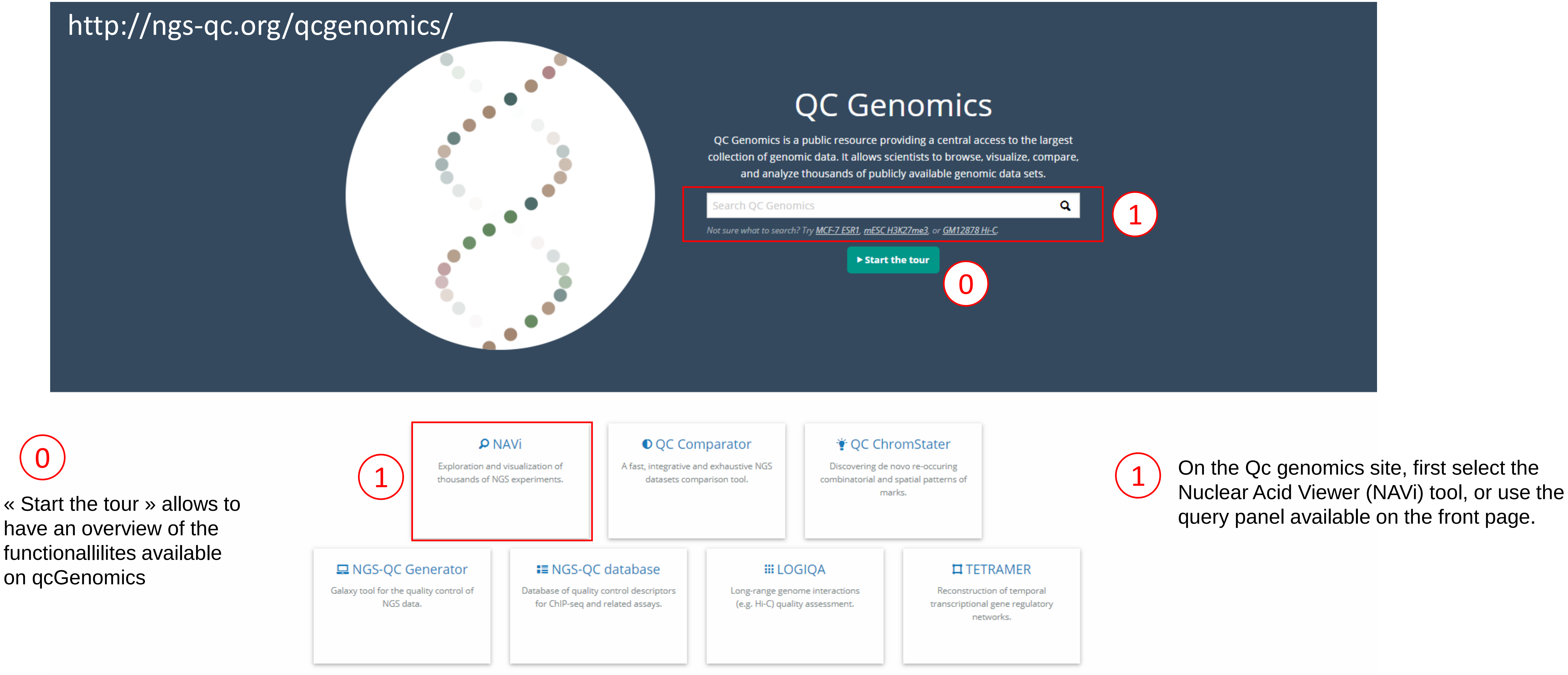

Supplementary Figure S1. Step by Step visualization of RNA-seq readouts on QC Genomics platform. The QC Genomics platform is mounted over the NGS-QC Generator database, hosting > 90 000 public datasets, covering ChIP-sequencing and enrichment related assays, including RNA-sequencing. QC Genomics, allows to retrieve datasets by the use of queries targeting the samples identifiers (e.g. GSM), cell/tissue types, target molecules (e.g. RNAseq, H3K4me3, etc), authors, etc. Retrieved datasets can be visualized on dedicated a genome browser to evaluate enrichment levels at defined genes or genomic coordinates, but also via multi profile comparison strategies (Global profile comparisons with QC Comparator or local combinatorial assays with QC ChromStater).

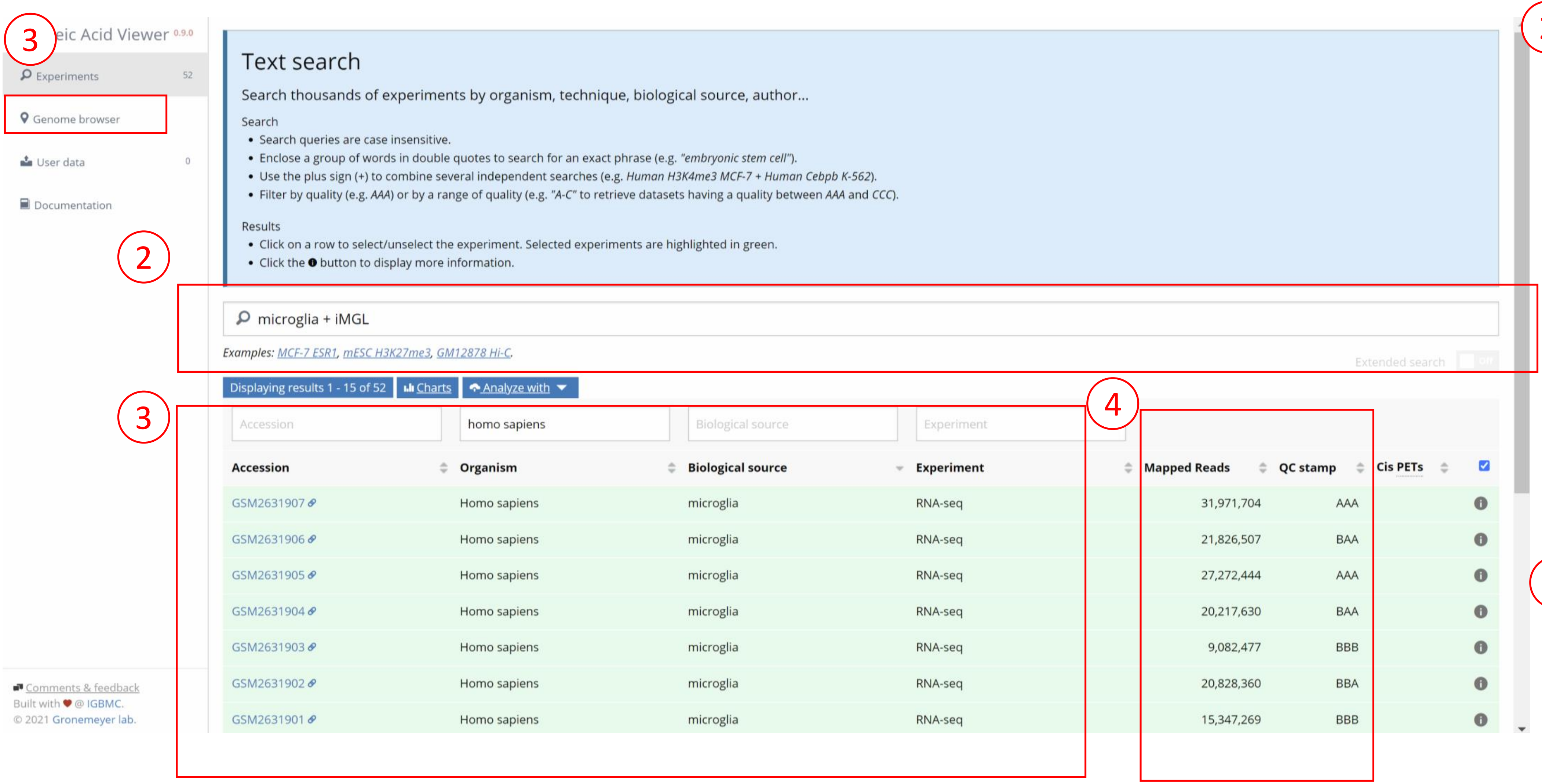

2

Data sets can be selected using broad terms ex : microglia on the upper reasearch bar,

The research can be refined using specicific terms in each sub section:
-Accession : GEO accession number

-Organism : Scientific name of the organism studied(Homo sapiens or mus musmuculus)

-Biological source : The type of cell (ex: «iMGL» or « microglia »)

-Experiement the type of sequencing Data (RNA-seq,ATAC-seq,…)

3

Datasets can be selected by perfoming a left click. The number of selected datasets is shown in the « experiment » Tab. Then, they can be visualized within the Genome browser tool.

4

For each dataset, information like their total number of mapped reads and a quality score (going from DDD to AAA, from worst to the best) is available. Further information is also available on a right click on the mouse.

Supplementary Figure S1. Step by Step visualization of RNA-seq readouts on QC Genomics platform. The QC Genomics platform is mounted over the NGS-QC Generator database, hosting > 90 000 public datasets, covering ChIP-sequencing and enrichment related assays, including RNA-sequencing. QC Genomics, allows to retrieve datasets by the use of queries targeting the samples identifiers (e.g. GSM), cell/tissue types, target molecules (e.g. RNAseq, H3K4me3, etc), authors, etc. Retrieved datasets can be visualized on dedicated a genome browser to evaluate enrichment levels at defined genes or genomic coordinates, but also via multi profile comparison strategies (Global profile comparisons with QC Comparator or local combinatorial assays with QC ChromStater).

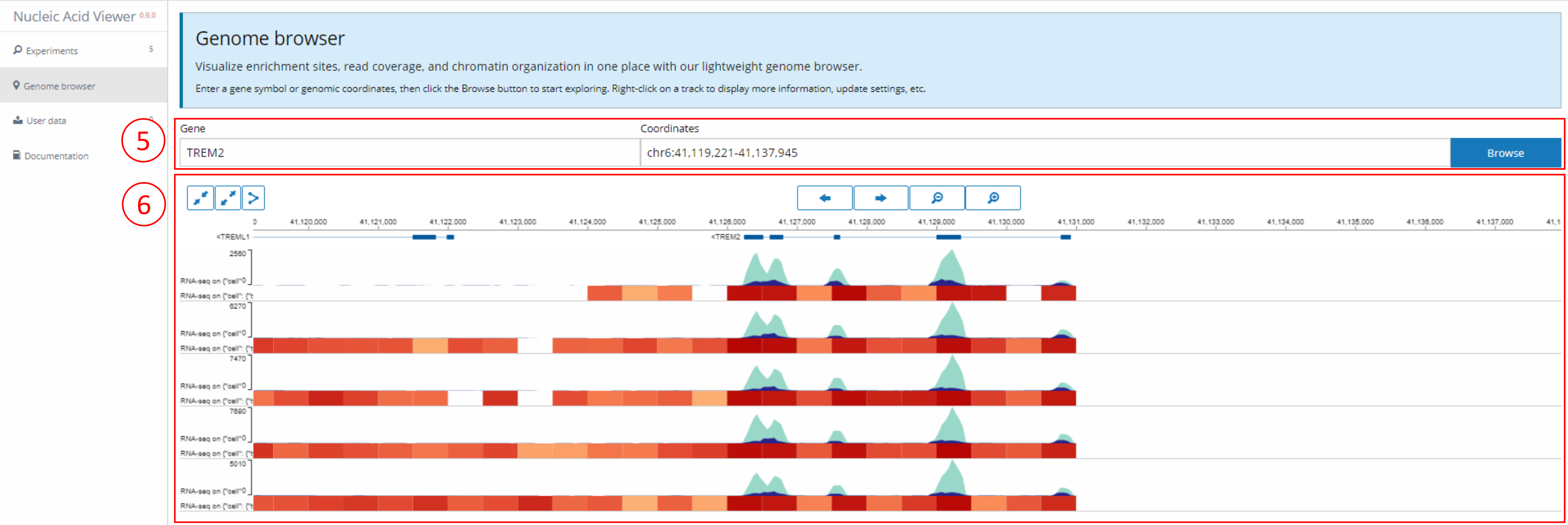

5 Select the gene or the chromosome coordinate of intrerest.

6 A quality enrichment graph associated to each of the queried datasets is displayed on a heatmap format. To display the read counts intensity, a right click on the mouse allows to access to a panel allowing to select either "coverage", or "coverage (all)" in case we want to display the read counts intensity for one or all datasets respectively.

Supplementary Figure S1. Step by Step visualization of RNA-seq readouts on QC Genomics platform. The QC Genomics platform is mounted over the NGS-QC Generator database, hosting > 90 000 public datasets, covering ChIP-sequencing and enrichment related assays, including RNA-sequencing. QC Genomics, allows to retrieve datasets by the use of queries targeting the samples identifiers (e.g. GSM), cell/tissue types, target molecules (e.g. RNAseq, H3K4me3, etc), authors, etc. Retrieved datasets can be visualized on dedicated a genome browser to evaluate enrichment levels at defined genes or genomic coordinates, but also via multi profile comparison strategies (Global profile comparisons with QC Comparator or local combinatorial assays with QC ChromStater).

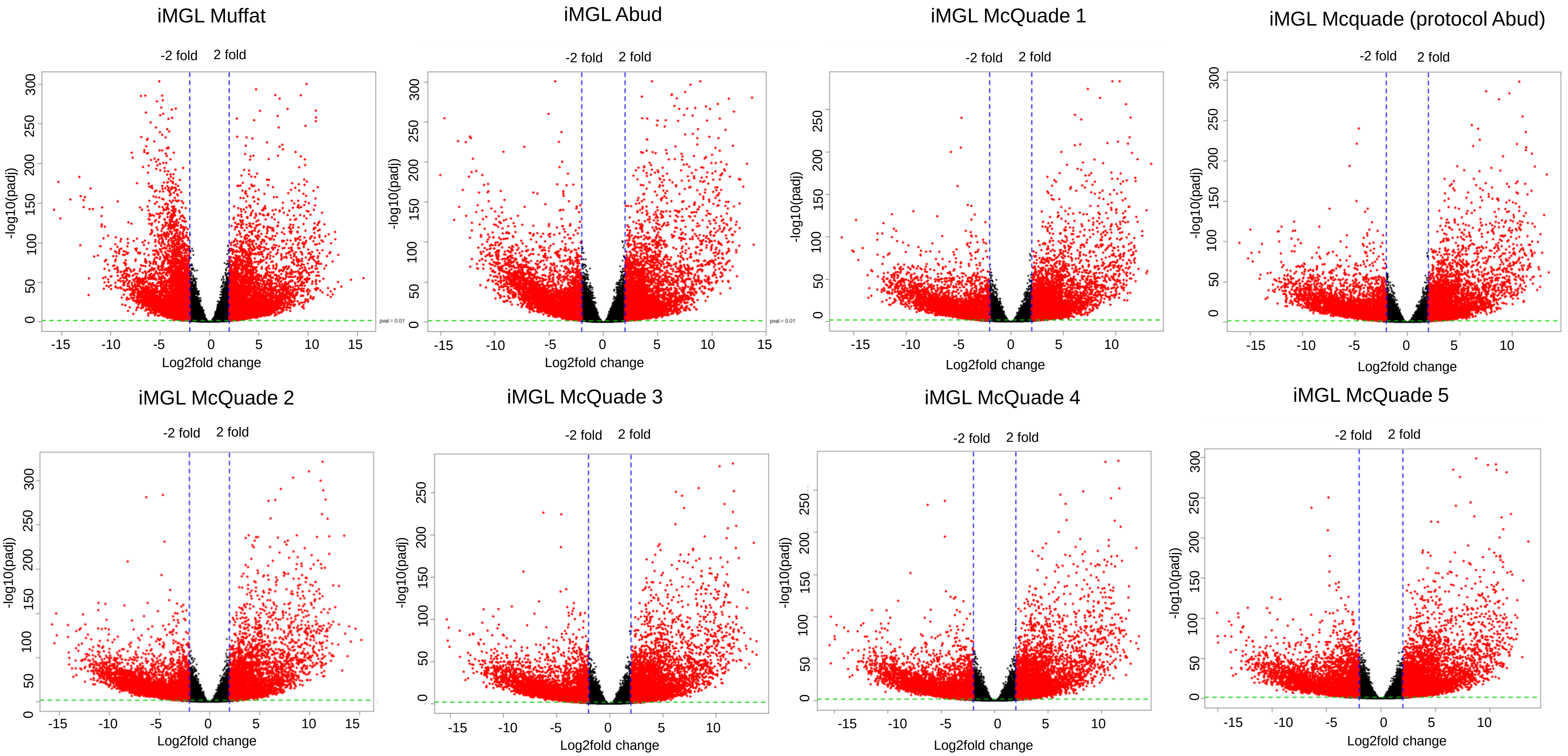

Supplementary Figure S2: Volcano plots revealing the differentially expressed genes (DEGs) in iMGL datasets generated by the various differentiation protocols. DEGs, defined by a log2 fold-change expression >2 (dashed vertical blue lines) and an adjustedP-value (padj) <0,01 (dashed horizontal green line), are displayed in red.

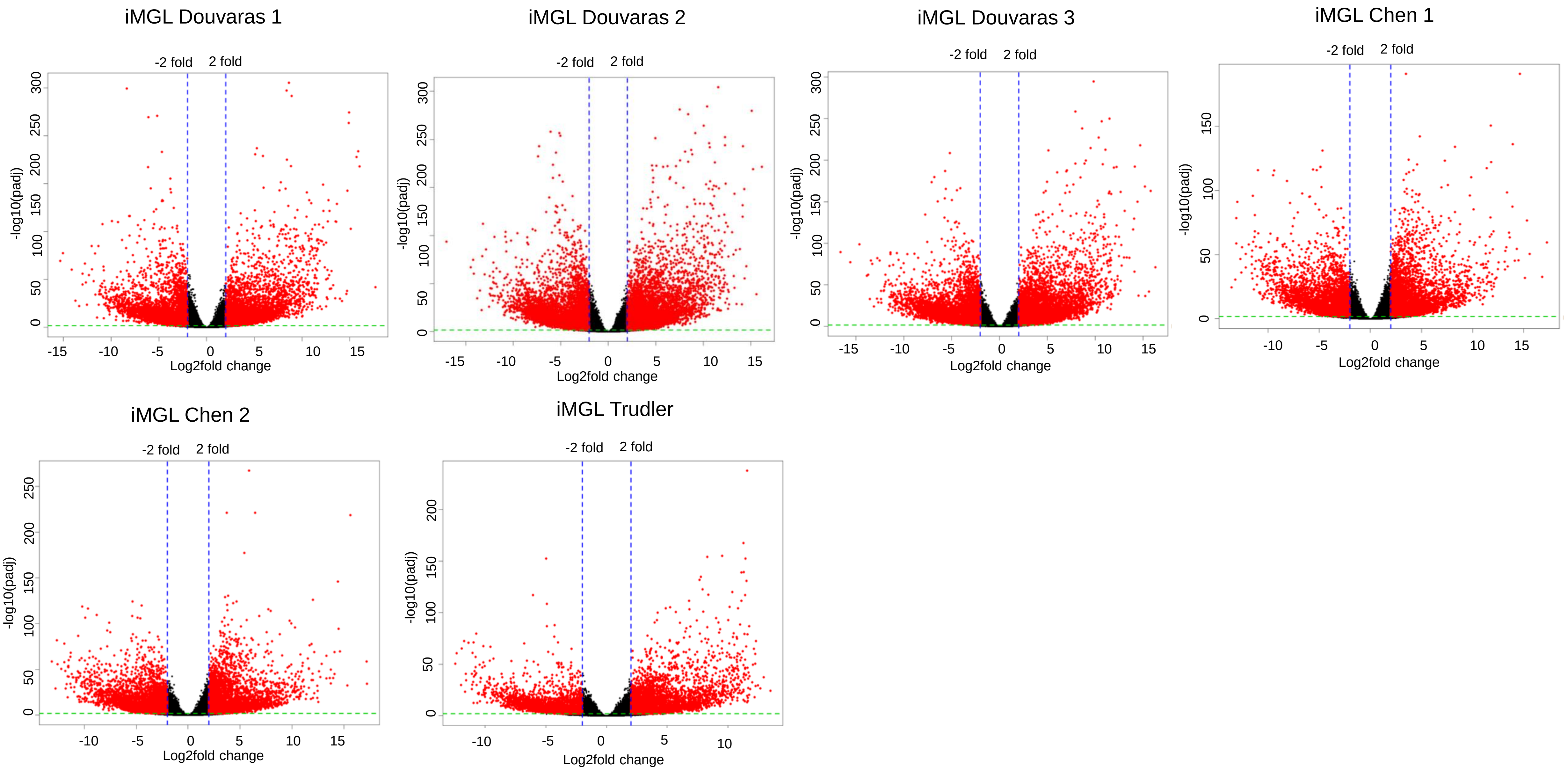

Supplementary Figure S2: Volcano plots revealing the differentially expressed genes (DEGs) in iMGL datasets generated by the various differentiation protocols. DEGs, defined by a log2 fold-change expression >2 (dashed vertical blue lines) and an adjustedP-value (padj) <0,01 (dashed horizontal green line), are displayed in red.

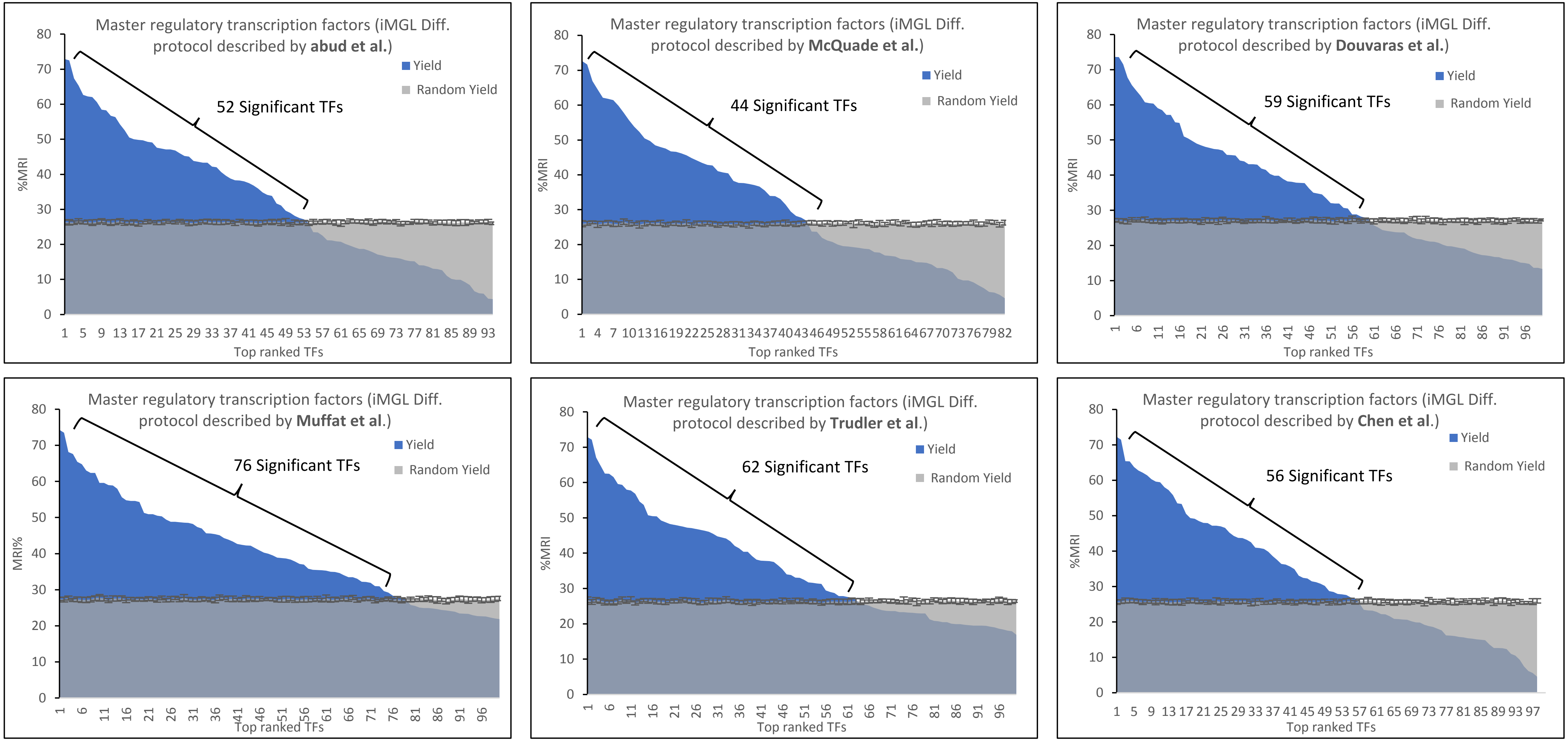

Supplementary Figure S3. Master regulatory Transcription factors predicted by TETRAMER for each of the differential expression data associated to each of the iMGL differentiation protocols. TETRAMER (Cholley et al; Nat. Sys. Biol & Appl; 2018) reconstruct a gene regulatory network (GRN) by integrating the differential expression data (iMGL vs iPS) and a collection of TF-target genes (TG) relationships issued from CAGE readouts (Marbach et al; Nat. Methods 2016). Then TETRAMER simulates TF transcription regulation over the whole GRN and evaluates the fraction of downstream TGs under its control, defined as the Master regulatory index (MRI). Each TF is ranked on the basis of their MRI and compared to a similar analysis over 10 randomized GRNs. “Yield” correspond to the MRI and “random yield” to the MRI assessed under the randomized conditions. Error bars over the randomized MRI reflects the fluctuation issued from the 10x randomization assays.
